# Supplementary material for: Experimental demonstration of spinor slow light
Source: Nat Commun. 2014 Nov 24;5:5542. doi: 10.1038/ncomms6542 (PMC4263133; doi:10.1038/ncomms6542)
Supplement: Supplementary Information — Supplementary Figures 1-4, Supplementary Notes 1-6. [file ncomms6542-s1.pdf]

Supplementary Figures

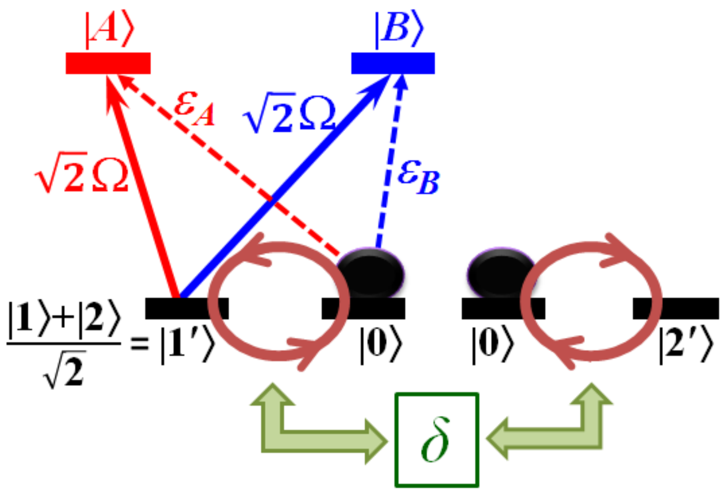

Supplementary Figure 1 | Transition diagram equivalent to the DT system at θ = 0. It consists of a double-Λ system and a two-ground-state system which couple with each other via the ground-state coherences.

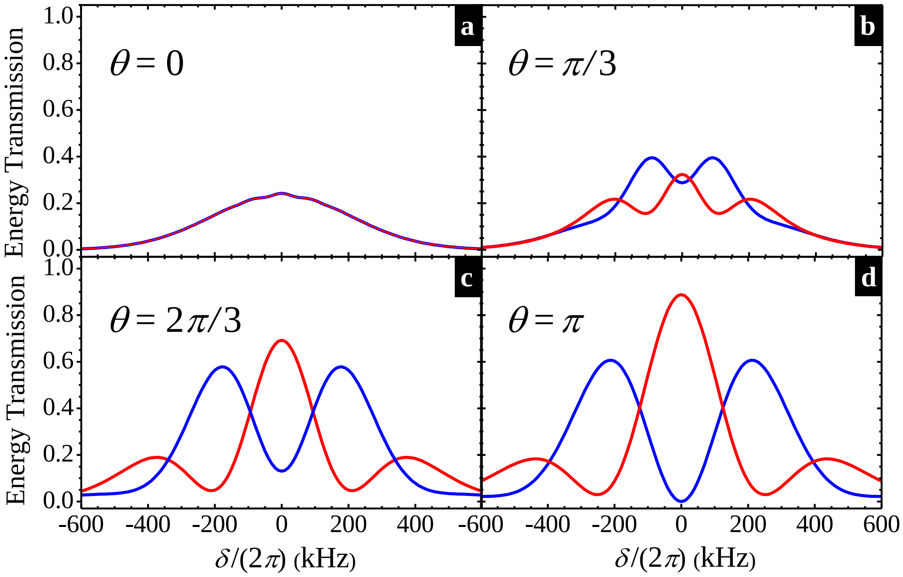

Supplementary Figure 2 | Theoretical predictions of the probe energy transmissions versus the detuning at different values of θ. Red and blue lines are the transmissions of ε_A and ε_B, respectively. In all the plots we set the calculation parameters to the experimental condition that α = 20, $|\Omega_{A1}| = |\Omega_{A2}| = |\Omega_{B1}| = |\Omega_{B2}| = 0.51\Gamma$, and only the probe pulse ε_A with the e^{-2} full width of 2.5 μs or $94\Gamma^{-1}$ is present in the input. The phase mismatch and ground-state coherence dephasing are not included in the calculation. In **a-d**, θ = 0, π/3, 2π/3 and π, respectively. The two lines completely overlap in **a**.

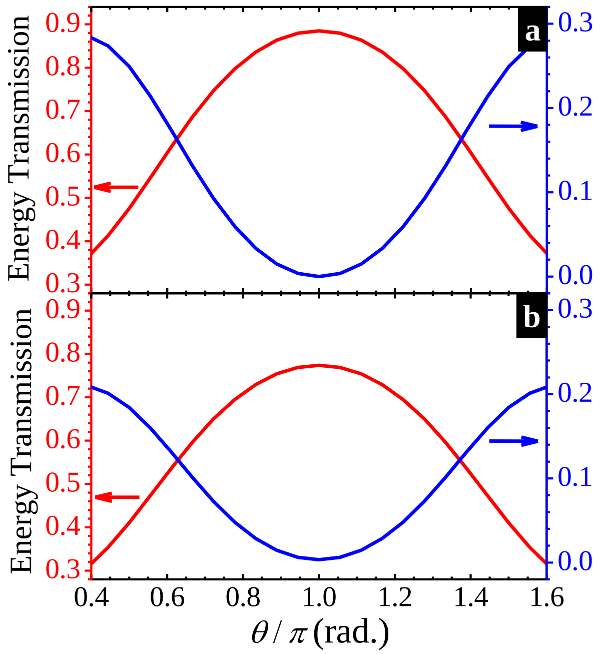

Supplementary Figure 3 | Theoretical predictions of the probe energy transmissions versus θ **at the zero detuning.** Red and blue lines are the transmissions of ε_A and ε_B, respectively. The calculation parameters of OD, coupling Rabi frequencies and input probe pulse are the same as those in Supplementary Figure 2. **a**, The phase mismatch and ground-state coherence dephasing are not included. **b**, We take $\Delta_k L$ = 0.6, γ_1 = 0 and γ_2 = $3.7\times10^{-3}\Gamma$ corresponding to the experimental situation.

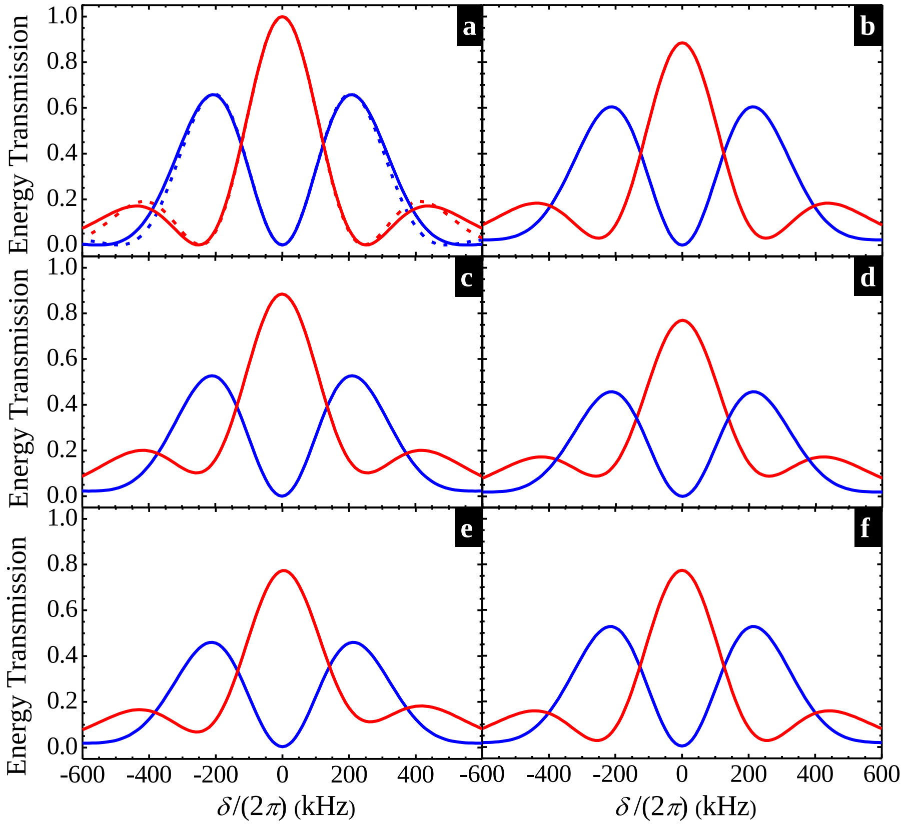

Supplementary Figure 4 | Theoretical predictions of the probe energy transmissions versus the detuning at $\theta = \pi$. Red and blue lines are the transmissions of ε_A and ε_B, respectively. Solid lines are the numerical predictions and dashed lines are the analytical results given by Eq. (6) of the main text. The calculation parameters of OD and coupling Rabi frequencies are the same as those in Supplementary Figure 2. Only probe ε_A is present in the input. In **a**, ε_A is a continuous wave; in **b-f**, ε_A is a pulse the same as that in Supplementary Figure 2. **a,b,** $\Delta_k L = 0$ and $\gamma_1 = \gamma_2 = 0$. **c**, $\Delta_k L = 0.6$ and $\gamma_1 = \gamma_2 = 0$. **d,** $\Delta_k L = 0.6$ and $\gamma_1 = \gamma_2 = 1.85\times10^{-3}\Gamma$. **e,** $\Delta_k L = 0.6$, $\gamma_1 = 0$ and $\gamma_2 = 3.7\times10^{-3}\Gamma$. **f,** $\Delta_k L = 0$, $\gamma_1 = 0$ and $\gamma_2 = 3.7\times10^{-3}\Gamma$.

Supplementary Notes

Supplementary Note 1: Analytical Solution of Continuous-Wave Spinor Slow Light

We consider the spinor slow light (SSL) forming when two probe beams are coupled with two atomic coherences in the double-tripod (DT) scheme of atom-light coupling. The time evolution of the SSL is described by the following equations:

$$\frac{1}{c}\frac{\partial}{\partial t}\begin{bmatrix}\varepsilon_A\\ \varepsilon_B\end{bmatrix}+\frac{\partial}{\partial z}\begin{bmatrix}\varepsilon_A\\ \varepsilon_B\end{bmatrix}+\begin{bmatrix}i\Delta_k/2 & 0\\ 0 & -i\Delta_k/2\end{bmatrix}\begin{bmatrix}\varepsilon_A\\ \varepsilon_B\end{bmatrix}=i\frac{\alpha\Gamma}{2L}\begin{bmatrix}\varepsilon_A\\ \varepsilon_B\end{bmatrix}, \tag{1}$$

$$\frac{\partial}{\partial t}\begin{bmatrix}\rho_A\\ \rho_B\end{bmatrix}=\frac{i}{2}\begin{bmatrix}\varepsilon_A\\ \varepsilon_B\end{bmatrix}+\frac{i}{2}\begin{bmatrix}\Omega_{A1} & \Omega_{A2}\\ \Omega_{B1} & \Omega_{B2}\end{bmatrix}\begin{bmatrix}\rho_1\\ \rho_1\end{bmatrix}-\frac{\Gamma}{2}\begin{bmatrix}\rho_A\\ \rho_B\end{bmatrix}, \tag{2}$$

$$\frac{\partial}{\partial t}\begin{bmatrix}\rho_1\\ \rho_2\end{bmatrix}=\frac{i}{2}\begin{bmatrix}\Omega_{A1}^* & \Omega_{B1}^*\\ \Omega_{A2}^* & \Omega_{B2}^*\end{bmatrix}\begin{bmatrix}\rho_A\\ \rho_B\end{bmatrix}+\begin{bmatrix}i\delta & 0\\ 0 & -i\delta\end{bmatrix}\begin{bmatrix}\rho_1\\ \rho_1\end{bmatrix}+\begin{bmatrix}-\gamma_1 & 0\\ 0 & -\gamma_2\end{bmatrix}\begin{bmatrix}\rho_1\\ \rho_1\end{bmatrix}, \tag{3}$$

where ε_A, ε_B, Ω_{A1}, Ω_{A2}, Ω_{B1} and Ω_{B2} are the Rabi frequencies of the probe and coupling fields driving the atomic transitions depicted in Fig. 1a of the main text, ρ_A (or ρ_B) is the coherence of the probe transition $|0\rangle\rightarrow|A\rangle$ (or $|0\rangle\rightarrow|B\rangle$), ρ_1 (or ρ_2) is the atomic ground-state coherence between $|0\rangle$ and $|1\rangle$ (or $|2\rangle$), Γ is the spontaneous decay rate equal to $2\pi\times 6$ MHz in our experiment, α and L are the optical density (OD) and length of the medium, δ is the two-photon detuning as illustrated in Fig. 1a of the main text, γ_1 (or γ_2) is the dephasing rate of the atomic coherence ρ_1 (or ρ_2), and $\Delta_k \equiv (-\vec{\mathbf{k}}_{pA}+\vec{\mathbf{k}}_{cA}-\vec{\mathbf{k}}_{cB}+\vec{\mathbf{k}}_{pB})\cdot\hat{\mathbf{z}}$ describes the effect of the phase mismatch. In the definition of Δ_k, $\vec{\mathbf{k}}_{pA}$ and $\vec{\mathbf{k}}_{pB}$ are the wave vectors of the probe fields ε_A and ε_B, and $\vec{\mathbf{k}}_{cA}$ and $\vec{\mathbf{k}}_{cB}$ are those of the coupling fields Ω_{A1} and Ω_{B1} (or Ω_{A2} and Ω_{B2}).The analytical expressions and numerical predictions in the paper and in the supplement are based on the above equations.

We focus on the case where the complex Rabi frequencies of the four coupling fields have the same amplitude of Ω. Thus, the complex Rabi frequency of the nth coupling field is $\Omega_n = \Omega e^{i\theta_n}$ with $n = A1$, $A2$, $B1$ or $B2$, where θ_n is the phase of coupling field. We define $\theta \equiv (\theta_{A1} - \theta_{A2}) - (\theta_{B1} - \theta_{B2})$ to be a relative phase among the four coupling fields. To simplify the analytical derivation, we take $\gamma_1 = 0 = \gamma_2$ and $\Delta_k = 0$. For the continuous waves, Eqs. (1)-(3) reduce to

$$\frac{\partial}{\partial z}\begin{bmatrix}\varepsilon_A\\ \varepsilon_B\end{bmatrix}=i\frac{\alpha\Gamma}{2L}\begin{bmatrix}\rho_A\\ \rho_B\end{bmatrix}, \tag{4}$$

$$0=\frac{i}{2}\begin{bmatrix}\varepsilon_A\\ \varepsilon_B\end{bmatrix}+\frac{i}{2}\begin{bmatrix}i\Omega & \Omega\\ \Omega & i\Omega\end{bmatrix}\begin{bmatrix}\rho_1\\ \rho_2\end{bmatrix}-\frac{\Gamma}{2}\begin{bmatrix}\rho_A\\ \rho_B\end{bmatrix}, \tag{5}$$

$$0=\frac{i}{2}\begin{bmatrix}-i\Omega & \Omega\\ \Omega & -i\Omega\end{bmatrix}\begin{bmatrix}\rho_A\\ \rho_B\end{bmatrix}+\begin{bmatrix}i\delta & 0\\ 0 & -i\delta\end{bmatrix}\begin{bmatrix}\rho_1\\ \rho_2\end{bmatrix}. \tag{6}$$

Here we set the phases of individual coupling fields (θ_{A1}, θ_{A2}, θ_{B1}, θ_{B2}) = ($\pi/2$, 0, 0, $\pi/2$) such that their relative phase is $\theta = \pi$. Other combinations, such as ($\pi/2$, $-\pi/2$, 0, 0), (π, 0, 0, 0), (0, 0, 0, π), etc., can also achieve the same result shown at the end of this section. To derive the solution of the above equations, we first make use of Eqs. (5) and (6) to eliminate (ρ_1, ρ_2), subsequently expressing (ρ_A, ρ_B) in terms of (ε_A, ε_B):

$$\begin{bmatrix}\rho_A\\ \rho_B\end{bmatrix}=\frac{i}{\Gamma}\begin{bmatrix}1 & -\beta\\ \beta & 1\end{bmatrix}^{-1}\begin{bmatrix}\varepsilon_A\\ \varepsilon_B\end{bmatrix}, \tag{7}$$

where

$$\beta \equiv \frac{\Omega^2}{\delta\Gamma}. \tag{8}$$

Consequently Eq. (4) becomes

$$\frac{\partial}{\partial z}\begin{bmatrix}\varepsilon_A \\ \varepsilon_B\end{bmatrix} = -\frac{\alpha}{2L}\frac{1}{1+\beta^2}\begin{bmatrix}1 & \beta \\ -\beta & 1\end{bmatrix}\begin{bmatrix}\varepsilon_A \\ \varepsilon_B\end{bmatrix}. \tag{9}$$

The matrix on the right-hand side of the above equation can be diagonalized by transforming the two probe fields (ε_A, ε_B) to new variables (ε_a, ε_b) via a unitary transformation

$$\begin{bmatrix}\varepsilon_a \\ \varepsilon_b\end{bmatrix} = \frac{1}{\sqrt{2}}\begin{bmatrix}1 & i \\ i & 1\end{bmatrix}\begin{bmatrix}\varepsilon_A \\ \varepsilon_B\end{bmatrix}. \tag{10}$$

Based on Eq. (9), the propagation equation for (ε_a, ε_b) and its solution are

$$\frac{\partial}{\partial z}\begin{bmatrix}\varepsilon_a \\ \varepsilon_b\end{bmatrix} = -\frac{\alpha}{2L}\frac{1}{1+\beta^2}\begin{bmatrix}1-i\beta & 0 \\ 0 & 1+i\beta\end{bmatrix}\begin{bmatrix}\varepsilon_a \\ \varepsilon_b\end{bmatrix} \tag{11}$$

and

$$\begin{bmatrix}\varepsilon_a(L) \\ \varepsilon_b(L)\end{bmatrix} = \exp\left(-\frac{\alpha}{2}\frac{1}{1+\beta^2}\right)\begin{bmatrix}\exp\left(i\frac{\alpha}{2}\frac{\beta}{1+\beta^2}\right) & 0 \\ 0 & \exp\left(-i\frac{\alpha}{2}\frac{\beta}{1+\beta^2}\right)\end{bmatrix}\begin{bmatrix}\varepsilon_a(0) \\ \varepsilon_b(0)\end{bmatrix}, \tag{12}$$

respectively. The transformed fields ε_a and ε_b represent the two normal modes inside the medium. Using the transformation Eq. (10), we obtain the output probe fields ε_A and ε_B as a function of their inputs:

$$\begin{bmatrix}\varepsilon_A(L) \\ \varepsilon_B(L)\end{bmatrix} = \exp\left(-\frac{\alpha}{2}\frac{1}{1+\beta^2}\right)\begin{bmatrix}\cos\left(\frac{\alpha}{2}\frac{\beta}{1+\beta^2}\right) & -\sin\left(\frac{\alpha}{2}\frac{\beta}{1+\beta^2}\right) \\ \sin\left(\frac{\alpha}{2}\frac{\beta}{1+\beta^2}\right) & \cos\left(\frac{\alpha}{2}\frac{\beta}{1+\beta^2}\right)\end{bmatrix}\begin{bmatrix}\varepsilon_A(0) \\ \varepsilon_B(0)\end{bmatrix}. \tag{13}$$

For $\beta \gg 1$ or $\Omega^2 \gg \delta\Gamma$, the solution simplifies to

$$\begin{bmatrix}\varepsilon_A(L) \\ \varepsilon_B(L)\end{bmatrix} = \exp\left(-\frac{2\phi^2}{\alpha}\right)\begin{bmatrix}\cos\phi & -\sin\phi \\ \sin\phi & \cos\phi\end{bmatrix}\begin{bmatrix}\varepsilon_A(0) \\ \varepsilon_B(0)\end{bmatrix}, \tag{14}$$

where

$$\phi = \frac{\alpha}{2}\frac{\delta\Gamma}{\Omega^2}. \tag{15}$$

The above two equations represent Eqs. (4) and (5) of the main text.

Supplementary Note 2: Double-Tripod System Equivalent to Two Coupled Λ Systems

Considering that the four coupling fields having the same amplitude, their relative phase θ equal to π, the dephasing rates and phase mismatch being negligible, Eqs. (2) and (3) become

$$\frac{\partial}{\partial t}\begin{bmatrix}\rho_A \\ \rho_B\end{bmatrix} = \frac{i}{2}\begin{bmatrix}\varepsilon_A \\ \varepsilon_B\end{bmatrix} + \frac{i}{2}\begin{bmatrix}\Omega & \Omega \\ \Omega & -\Omega\end{bmatrix}\begin{bmatrix}\rho_1 \\ \rho_2\end{bmatrix} - \frac{\Gamma}{2}\begin{bmatrix}\rho_A \\ \rho_B\end{bmatrix}, \tag{16}$$

$$\frac{\partial}{\partial t}\begin{bmatrix}\rho_1 \\ \rho_2\end{bmatrix} = \frac{i}{2}\begin{bmatrix}\Omega & \Omega \\ \Omega & -\Omega\end{bmatrix}\begin{bmatrix}\rho_A \\ \rho_B\end{bmatrix} + \begin{bmatrix}i\delta & 0 \\ 0 & -i\delta\end{bmatrix}\begin{bmatrix}\rho_1 \\ \rho_2\end{bmatrix}. \tag{17}$$

For convenience here we set (θ_{A1}, θ_{A2}, θ_{B1}, θ_{B2}) = (0, 0, 0, π). Other combinations, such as (π/2, 0, 0, π/2), (π/2, $-\pi$/2, 0, 0), (π, 0, 0, 0), etc., can also achieve the same conclusion shown at the end of this section. We introduce a unitary transformation U given by

$$U = \frac{1}{\sqrt{2}}\begin{bmatrix} 1 & 1 \\ 1 & -1 \end{bmatrix}. \tag{18}$$

and insert it in Eqs. (16) and (17) in the following way:

$$\frac{\partial}{\partial t}\begin{bmatrix} \rho_A \\ \rho_B \end{bmatrix} = \frac{i}{2}\begin{bmatrix} \varepsilon_A \\ \varepsilon_B \end{bmatrix} + \frac{i}{2}\left(\begin{bmatrix} \Omega & \Omega \\ \Omega & -\Omega \end{bmatrix} U^{-1}\right)\left(U\begin{bmatrix} \rho_1 \\ \rho_2 \end{bmatrix}\right) - \frac{\Gamma}{2}\begin{bmatrix} \rho_A \\ \rho_B \end{bmatrix}, \tag{19}$$

$$\frac{\partial}{\partial t}\left(U\begin{bmatrix} \rho_1 \\ \rho_2 \end{bmatrix}\right) = \frac{i}{2}\left(U\begin{bmatrix} \Omega & \Omega \\ \Omega & -\Omega \end{bmatrix}\right)\begin{bmatrix} \rho_A \\ \rho_B \end{bmatrix} + \left(U\begin{bmatrix} i\delta & 0 \\ 0 & -i\delta \end{bmatrix} U^{-1}\right)\left(U\begin{bmatrix} \rho_1 \\ \rho_2 \end{bmatrix}\right). \tag{20}$$

It is convenient to define two new variables $\rho_{1'}$ and $\rho_{2'}$ as

$$\begin{bmatrix} \rho_{1'} \\ \rho_{2'} \end{bmatrix} = U\begin{bmatrix} \rho_1 \\ \rho_2 \end{bmatrix}. \tag{21}$$

The new variable $\rho_{1'}$ (or $\rho_{2'}$) represents the ground-state coherence between the states $|1'\rangle$ (or $|2'\rangle$) and $|0\rangle$, where $|1'\rangle$ and $|2'\rangle$ are the following superpositions of the original ground states $|1\rangle$ and $|2\rangle$:

$$|1'\rangle = \frac{1}{\sqrt{2}}\left(|1\rangle + |2\rangle\right), \tag{22}$$

$$|2'\rangle = \frac{1}{\sqrt{2}}\left(|1\rangle - |2\rangle\right). \tag{23}$$

Finally, Eqs. (19) and (20) become

$$\frac{\partial}{\partial t}\begin{bmatrix} \rho_A \\ \rho_B \end{bmatrix} = \frac{i}{2}\begin{bmatrix} \varepsilon_A \\ \varepsilon_B \end{bmatrix} + \frac{i}{2}\begin{bmatrix} \sqrt{2}\Omega & 0 \\ 0 & \sqrt{2}\Omega \end{bmatrix}\begin{bmatrix} \rho_{1'} \\ \rho_{2'} \end{bmatrix} - \frac{\Gamma}{2}\begin{bmatrix} \rho_A \\ \rho_B \end{bmatrix}, \tag{24}$$

$$\frac{\partial}{\partial t}\begin{bmatrix} \rho_{1'} \\ \rho_{2'} \end{bmatrix} = \frac{i}{2}\begin{bmatrix} \sqrt{2}\Omega & 0 \\ 0 & \sqrt{2}\Omega \end{bmatrix}\begin{bmatrix} \rho_A \\ \rho_B \end{bmatrix} + \begin{bmatrix} 0 & i\delta \\ i\delta & 0 \end{bmatrix}\begin{bmatrix} \rho_{1'} \\ \rho_{2'} \end{bmatrix}. \tag{25}$$

According to the above equations, there are two EIT systems. One consists of ε_A, ρ_A and $\rho_{1'}$, the other being made of ε_B, ρ_B and $\rho_{2'}$. The coupling fields in both systems have the same Rabi frequency of $\sqrt{2}\Omega$. In Eqs. (24) and (25), the only matrix with non-zero off-diagonal terms is

$$\begin{bmatrix} 0 & i\delta \\ i\delta & 0 \end{bmatrix}.$$

It indicates that the interaction between the two ground-state coherences $\rho_{1'}$ and $\rho_{2'}$ (i.e. the coupling between the two EIT systems) is induced by the detuning δ. Therefore, the DT system is equivalent to the two coupled EIT systems as depicted in Fig. 1b of the main text.

Supplementary Note 3: Degenerate Double-Tripod System

The DT system becomes degenerate as the pair of the coupling fields in each tripod have the same complex Rabi frequency, i.e. the relative phase θ = 0. Under this special condition, the DT system will be shown to be equivalent to a double-Λ system and no oscillation can occur between the two probe fields. For convenience but without loss of generality, the four coupling fields have the same complex Rabi frequency of Ω. Considering the dephasing rates and phase mismatch to be negligible, Eqs. (2) and (3) become

$$\frac{\partial}{\partial t}\begin{bmatrix}\rho_A\\ \rho_B\end{bmatrix}=\frac{i}{2}\begin{bmatrix}\varepsilon_A\\ \varepsilon_B\end{bmatrix}+\frac{i}{2}\begin{bmatrix}\Omega & \Omega\\ \Omega & \Omega\end{bmatrix}\begin{bmatrix}\rho_1\\ \rho_2\end{bmatrix}-\frac{\Gamma}{2}\begin{bmatrix}\rho_A\\ \rho_B\end{bmatrix}, \tag{26}$$

$$\frac{\partial}{\partial t}\begin{bmatrix}\rho_1\\ \rho_2\end{bmatrix}=\frac{i}{2}\begin{bmatrix}\Omega & \Omega\\ \Omega & \Omega\end{bmatrix}\begin{bmatrix}\rho_A\\ \rho_B\end{bmatrix}+\begin{bmatrix}i\delta & 0\\ 0 & -i\delta\end{bmatrix}\begin{bmatrix}\rho_1\\ \rho_2\end{bmatrix}. \tag{27}$$

Using the two variables $\rho_{1'}$ and $\rho_{2'}$ defined in Eq. (21), we rewrite the above equations as

$$\frac{\partial}{\partial t}\begin{bmatrix}\rho_A\\ \rho_B\end{bmatrix}=\frac{i}{2}\begin{bmatrix}\varepsilon_A\\ \varepsilon_B\end{bmatrix}+\frac{i}{2}\begin{bmatrix}\sqrt{2}\Omega & 0\\ \sqrt{2}\Omega & 0\end{bmatrix}\begin{bmatrix}\rho_{1'}\\ \rho_{2'}\end{bmatrix}-\frac{\Gamma}{2}\begin{bmatrix}\rho_A\\ \rho_B\end{bmatrix}, \tag{28}$$

$$\frac{\partial}{\partial t}\begin{bmatrix}\rho_{1'}\\ \rho_{2'}\end{bmatrix}=\frac{i}{2}\begin{bmatrix}\sqrt{2}\Omega & \sqrt{2}\Omega\\ 0 & 0\end{bmatrix}\begin{bmatrix}\rho_A\\ \rho_B\end{bmatrix}+\begin{bmatrix}0 & i\delta\\ i\delta & 0\end{bmatrix}\begin{bmatrix}\rho_{1'}\\ \rho_{2'}\end{bmatrix}. \tag{29}$$

In Eq. (28), both ρ_A and ρ_B (or ε_A and ε_B) couple to the same ground-state coherence $\rho_{1'}$ as indicated by the term

$$\frac{i}{2}\begin{bmatrix}\sqrt{2}\Omega & 0\\ \sqrt{2}\Omega & 0\end{bmatrix}\begin{bmatrix}\rho_{1'}\\ \rho_{2'}\end{bmatrix}.$$

In Eq. (29), the ground-state coherence $\rho_{2'}$ does not interact with ρ_A and ρ_B directly as indicated by the term

$$\frac{i}{2}\begin{bmatrix}\sqrt{2}\Omega & \sqrt{2}\Omega\\ 0 & 0\end{bmatrix}\begin{bmatrix}\rho_A\\ \rho_B\end{bmatrix},$$

but the two ground-states coherences are coupled via the detuning δ as indicated by the term

$$\begin{bmatrix}0 & i\delta\\ i\delta & 0\end{bmatrix}\begin{bmatrix}\rho_{1'}\\ \rho_{2'}\end{bmatrix}.$$

Therefore, the DT system at $\theta = 0$ can be decomposed into two coupled subsystems. One is the double-Λ system in which the two probe fields share the common coherence $\rho_{1'}$. The other is the system only consisting of two ground states in which no light appears but the coherence $\rho_{2'}$ exists. Supplementary Figure 1 depicts the transition diagram equivalent to the DT system at $\theta = 0$. Because the two probe fields share the common ground-state coherence and interact with the medium in a similar way, their outputs always behave the same which will be demonstrated in the next section.

Supplementary Note 4: Oscillation Behaviors at Different Relative Phases of the Coupling Fields

The SSL oscillations can also be observed for the relative phase θ of the coupling fields other than π. Increasing the deviation of θ from π makes the oscillation less prominent, as the temporal profile of the probe field is a pulse. Finally, for a maximum deviation at $\theta = 0$, the DT system becomes equivalent to the double-Λ system as discussed in the previous section, and no oscillation can occur between the probe fields.

Supplementary Figures 2a-2d show the theoretical predictions of the probe energy transmission as a function of the detuning δ at different values of θ. The predictions were numerically calculated from Eqs. (1)-(3) by taking the parameters of input probe pulse width, optical density and coupling Rabi frequencies used in the experiment. The phase mismatch Δ_k and the ground-state dephasing rates γ_1 and γ_2 are set to zero in the calculation. The prediction in Supplementary Figure 2a shows the two probe pulses always have the same output energy (also temporal shape) at $\theta = 0$. This is the expected outcome of the double-Λ system with the same coupling Rabi frequency in each of the two constituent Λ systems. As demonstrated by Supplementary Figures 2b-2d, the condition of $\theta = \pi$ gives the maximum contrast or difference between two output probe fields around $\delta = 0$. For this reason, $\theta = \pi$ was chosen in the experiment.

Supplementary Note 5: Method of Setting the Relative Phase of the Coupling Fields to π

The relative phase of the coupling fields θ can be made equal to π directly, when the probe field is a pulse instead of a continuous wave. The theoretical predictions of the two output probe energy transmissions versus θ at the detuning $\delta = 0$ shown in Supplementary Figures 3a and 3b illustrate the idea. They were numerically calculated from Eqs. (1)-(3) with the parameters of input probe pulse width, optical density and coupling Rabi frequencies used in the experiment. As only the probe ε_A is present at the input of the medium, the probe ε_B will not be generated at the output if $\theta = \pi$. A larger deviation from π in θ causes larger output energy of ε_B. The inclusion of the phase mismatch and ground-state coherence dephasing existing in the experimental system do not change the behavior of ε_B's output energy versus θ.

Experimentally, we moved the position of the prism shown in Fig. 1c of the main text and measured ε_B's output energy at $\delta = 0$. According to its definition, θ is the phase difference between the beat note of Ω_{A1} plus Ω_{A2} and that of Ω_{B1} plus Ω_{B2}. The frequency difference between Ω_{A1} and Ω_{A2} (or between Ω_{B1} and Ω_{B2}) is about 6.8 GHz, corresponding to the beat-note wavelength of 4.4 cm. The prism position can change the optical path length of Ω_{A1} plus Ω_{A2} and, thus, adjust θ. By minimizing ε_B's output, we were able to properly set $\theta = \pi$.

Supplementary Note 6: Nonzero Minima and Asymmetry in the Oscillation Phenomenon

Let us now discuss the discrepancy between the observed SSL output and the oscillation phenomenon predicted by Eq. (6) of the main text. There are two major features in the discrepancy. The minima of the probe field ε_A are not completely zero, and the probe transmissions are asymmetric at the positive and negative detunings. Supplementary Figure 4a shows the predictions numerically calculated from Eqs. (1)-(3) for a continuous-wave input probe. The numerical predictions are in a good agreement with the analytical expression given by Eq. (6) of the main text except at large $|\delta|$ where the condition $\Omega^2 \gg \delta\Gamma$ is not held. When the input probe is a Gaussian pulse with the width used in the experiment, Supplementary Figure 4b clearly shows ε_A's minima become about 3%. Supplementary Figures 4c-4f all consider the input probe is a pulse. When the amount of $\Delta_k L$ existing in the experiment is added to the calculation, the total output energy is affected very little but ε_A's minima further increase to 10% as shown in Supplementary Figure 4c. Hence, the nonzero minima are caused by the existence of the phase mismatch Δ_k and the finite frequency bandwidth of the input probe pulse.

We further include the ground-state coherence dephasing in the calculation. As the two dephasing rates γ_1 and γ_2 of the coherences ρ_1 and ρ_2 are the same, Supplementary Figure 4d shows the total output energy is significantly decreased. So far, the spectra are all symmetric with respect to $\delta = 0$. By setting $\gamma_1 \neq \gamma_2$, we can clearly see that ε_B's spectrum is still symmetric but ε_A's spectrum becomes asymmetric as shown in Supplementary Figure 4e. By setting $\Delta_k = 0$ and $\gamma_1 \neq \gamma_2$, ε_A's spectrum becomes symmetric again as shown in Supplementary Figure 4f. Therefore, the asymmetry is caused by the combination of $\Delta_k \neq 0$ and $\gamma_1 \neq \gamma_2$.
